# Changes and Adaptations: How University Students Self-Regulate Their Online Learning During the COVID-19 Pandemic

**DOI:** 10.3389/fpsyg.2021.642593

**Published:** 2021-04-23

**Authors:** Felicitas Biwer, Wisnu Wiradhany, Mirjam oude Egbrink, Harm Hospers, Stella Wasenitz, Walter Jansen, Anique de Bruin

**Affiliations:** ^1^Department of Educational Development & Research, School of Health Professions Education (SHE), Faculty of Health, Medicine and Life Sciences, Maastricht University, Maastricht, Netherlands; ^2^Department of Psychology, Faculty of Humanities, Bina Nusantara University, West Jakarta, Indonesia; ^3^Department of Physiology, Faculty of Health, Medicine and Life Sciences, School of Health Professions Education (SHE), Maastricht University, Maastricht, Netherlands; ^4^EDLAB, Maastricht University Institute for Education Innovation, Maastricht, Netherlands

**Keywords:** COVID-19, self-regulated learning, resource-management strategies, emergency remote learning, cluster analysis, higher education

## Abstract

During the COVID-19 (coronavirus disease 2019) pandemic, universities had to shift from face-to-face to emergency remote education. Students were forced to study online, with limited access to facilities and less contact with peers and teachers, while at the same time being exposed to more autonomy. This study examined how students adapted to emergency remote learning, specifically focusing on students’ resource-management strategies using an individual differences approach. One thousand eight hundred university students completed a questionnaire on their resource-management strategies and indicators of (un)successful adaptation to emergency remote learning. On average, students reported being less able to regulate their attention, effort, and time and less motivated compared to the situation before the crisis started; they also reported investing more time and effort in their self-study. Using a *k*-means cluster analysis, we identified four adaptation profiles and labeled them according to the reported changes in their resource-management strategies: the overwhelmed, the surrenderers, the maintainers, and the adapters. Both the overwhelmed and surrenderers appeared to be less able to regulate their effort, attention, and time and reported to be less motivated to study than before the crisis. In contrast, the adapters appreciated the increased level of autonomy and were better able to self-regulate their learning. The resource-management strategies of the maintainers remained relatively stable. Students’ responses to open-answer questions on their educational experience, coded using a thematic analysis, were consistent with the quantitative profiles. Implications about how to support students in adapting to online learning are discussed.

## Introduction

In spring 2020, universities across the globe had to shift their face-to-face education to online because of the coronavirus disease 2019 (COVID-19) outbreak. From one day to the next, university students were forced to study online, either in isolation, in student housing, or in family settings—exposing them to many distractions. Furthermore, anxiety and uncertainty about the unprecedented situation may have caused additional stress (Son et al., 2020). Altogether, this sudden change to online education, termed *emergency remote education*, and subsequently *emergency remote learning* (Hodges et al., 2020), posed many challenges to students. At the same time, the shift to emergency remote education gave students more autonomy, and it increased the need for taking control of their own learning process (Dillon and Greene, 2003; Garrison, 2003). As emergency remote education is different from regular online education, it is important to understand whether and how students adapted to emergency remote learning. The aim of the present study was to gain insight into university students’ adaptation to emergency remote learning, specifically focusing on aspects of their self-regulated learning. Moreover, we wanted to know if students differed in their adaptation approach in order to gain insights in how to provide individual support.

### Self-Regulated Learning During Emergency Remote Learning

In both on-site and online higher education environments, university students already have a considerable amount of autonomy. They need to plan, monitor, and control their own learning process during self-study and thus engage in self-regulated learning (Nelson and Narrens, 1990; Zimmerman, 2002). Three main categories of learning strategies can be differentiated in self-regulated learning: cognitive, metacognitive, and resource-management strategies (Duncan and McKeachie, 2005; Panadero, 2017). Cognitive and metacognitive strategies are used to process information and monitor and control one’s understanding, whereas resource-management strategies are used to create optimal learning conditions. Resource-management strategies refer to managing external resources, as in seeking for help or organizing one’s workplace, as well as to managing and regulating internal resources, such as effort regulation, time management, attentional regulation, and motivation (Dresel et al., 2015).

Given the sudden shift to emergency remote education at the start of the COVID-19 pandemic, combined with external stress factors, such as uncertainty about the situation, distraction at home and reduced social interaction (Son et al., 2020), as well as higher levels of autonomy, resource-management strategies may have played an important role in adapting successfully to emergency remote education. Students probably already adopted effective cognitive and metacognitive strategies because of their experience of independence during higher education, but they had to quickly adapt these strategies to apply them in the new situation (Wood et al., 2005). Effective resource-management strategies have been shown to have a positive link to cognitive, emotional, and motivational aspects of learning. In relation to cognitive factors, resource-management strategies, specifically effort regulation, time management, and attentional regulation (concentration and dealing with distraction), were positively associated with academic performance in both face-to-face (Richardson et al., 2012) and online learning environments (Broadbent and Poon, 2015; Broadbent, 2017). With regard to emotional factors, facets of resource-management strategies, such as the organization of academic study time and motivation to invest effort in studying, are negatively affected by negative emotions (Mega et al., 2014). Furthermore, resource-management strategies, such as effort regulation and time management as well as intrinsic motivation, have been found to be positively associated with academic adjustment (van Rooij et al., 2018), which might be an indicator of their importance in adapting to emergency remote learning.

Adapting to higher levels of autonomy and successfully applying these resource-management strategies are, however, no easy feat for many students. A recent systematic review showed that students who choose to participate in online (blended) education struggle to use these strategies adequately; they experience self-regulation, motivational control, help seeking, and their technological competencies as main challenges (Rasheed et al., 2020). During the COVID-19 pandemic, students might experience similar but also additional challenges. Other than regular online education, emergency remote learning during COVID-19 involves learning in suboptimal spaces and isolation, putting a higher load on learners’ resource management. Because of not having access to their regular study environment such as the library or other university buildings, students might have trouble to find a quiet study space, which potentially influences their attentional regulation (Dabbish et al., 2011). In addition, compared to regular online education, the change to emergency remote learning during COVID-19 was not voluntary, which may have had a negative influence on students’ study motivation (Hsu et al., 2019). Furthermore, given the sudden shift to online education, students may not have had access to all technical resources (e.g., stable internet connection) or support from teaching staff and peers. Given the uniqueness of the situation, it is important to build an understanding on whether and how students were able to adapt their resource-management strategies when confronted with emergency remote learning.

### Unraveling Individual Differences in Adaptation to Emergency Remote Education

There is increasing evidence that self-regulatory processes, including resource-management strategies, vary across individuals (Barnard-Brak et al., 2010; Dörrenbächer and Perels, 2016). Additionally, students with better self-regulated learning skills have been shown to have higher academic performance (Kitsantas et al., 2008; Barnard-Brak et al., 2010; Broadbent and Fuller-Tyszkiewicz, 2018) and better self-regulated learning intervention outcomes (Dörrenbächer and Perels, 2016). Given the individual differences in self-regulated learning, students might respond differently in the situation of emergency remote learning: some students might find it difficult to concentrate, whereas others might double their efforts to cope with the new environment (Usher and Schunk, 2018). This is in line with the social cognitive framework on self-regulation, which suggests self-regulated learning as an interaction between personal, behavioral, and environmental factors (Usher and Schunk, 2018). Learning is situated in specific contexts, and self-regulatory processes may differ depending on the context (Boekaerts and Niemivirta, 2000; Pintrich, 2000; Efklides, 2011). For example, Broadbent and Fuller-Tyszkiewicz (2018) examined profiles in self-regulated learning for online and blended learning students. The authors uncovered five profiles of self-regulation, with online learners being more likely to belong to more adaptive profiles. Students with the highest grades had also the highest levels of time management, effort regulation, and motivation, indicating that individual approaches to learning impact performance. Uncovering subgroups of students, e.g., those who struggle significantly and those who are able to adapt more easily, and understanding different profiles of adaptation during emergency remote learning could yield important insights in how to provide tailored support to students (Barnard-Brak et al., 2010; Broadbent and Fuller-Tyszkiewicz, 2018).

In the current study, we examined how and to what extent university students adapted their resource-management strategies during emergency remote learning because of the COVID-19 pandemic. Using a mixed-method approach, we first investigated to what extent the sudden shift from face-to-face to emergency remote education influenced students’ self-regulated learning, with a specific focus on their resource-management strategies: their effort and attentional regulation, motivation, time management, and time and effort investment. Specific questionnaires are available to assess students’ online self-regulated learning in the contexts of MOOCs or blended learning environments. These questionnaires are adaptations of classical self-regulated learning questionnaires for on-site education to online education (Barnard et al., 2009; Jansen et al., 2017). Because of the context specificity of self-regulated learning and unique characteristics of emergency remote learning, we decided to take changes in context into account when measuring how students adapted to emergency remote learning. We therefore modified existing online self-regulated learning questionnaires (Barnard et al., 2009; Jansen et al., 2017) in order to measure changes in students’ time management, effort regulation, attentional regulation, motivation, and effort and time investment. We expected these dimensions to be most influenced by emergency remote education.

Our second goal was to examine whether students adapted differently to emergency remote learning using a person-centered approach. In contrast to variable-centered approaches that assume that relationships between self-regulatory processes observed at group level are representative for the whole sample, person-centered approaches assume potential differences between subgroups of students (Laursen and Hoff, 2006). Here, we explored whether potential differences between subgroups of students were related to their general experience with education before and after the shift to online learning, engagement, and well-being as indicators of (un)successful adaptation to the situation. Third, we investigated students’ experiences as difficulties and benefits of emergency remote learning, by examining their reactions to open-answer questions on this topic. By gaining insight into the different difficulties, but also potential benefits that students experienced, we aim to further inform and generate ideas about how to support students. In summary, we address the following research questions:

(1) How did the sudden shift from face-to-face to emergency remote education influence university students’ self-regulated learning, focusing on their resource-management strategies?
(2) Did students adapt differently to emergency remote learning?
(3) What are the main difficulties and benefits of emergency remote learning for students?

## Materials and Methods

### Setting and Participants

In March 2020, the Dutch government announced measures to stop the spread of COVID-19, among others by forcing all universities to shift all their education from face-to-face to online. At Maastricht University, education is based on problem-based learning (PBL), applying four core learning principles: constructive, collaborative, contextual, and self-directed learning (Dolmans et al., 2005). Students work on authentic, real-world cases in small tutorial groups consisting of 10–15 students. A tutor moderates the tutorial sessions as facilitator. The academic year is usually divided into six course periods of 8 or 4 weeks, each period focusing on a specific theme. The shift to online education occurred at the end of course period 4.

In May 2020, all bachelor’s and master’s degree students at Maastricht University (*N* = 17,182) were invited to complete an online questionnaire about their experiences during emergency remote learning. In total, 1,817 students (mean_*age*_ = 21.3 years, 68% females) participated, which corresponds to a response rate of 10.5%. The sample included 1,543 bachelor’s degree students and 274 master’s degree students from all six faculties: Faculty of Science and Engineering (25%), School of Business and Economics (23%), Faculty of Health, Medicine, and Life Sciences (20%), Faculty of Law (14%), Faculty of Arts and Social Sciences (9%), and Faculty of Psychology and Neurosciences (9%).

### Measures

#### Demographic Survey

Respondents were asked to report their age, gender, program level (bachelor’s or master’s degree), faculty of study, study program, and whether they were a regular Maastricht University student or an exchange student from another university.

#### Resource-Management Strategies

We composed a questionnaire (17 items) that assesses how the new situation influenced students’ use of resource-management strategies, based on existing questionnaires on online self-regulated learning (Barnard et al., 2009; Jansen et al., 2017). The adapted theoretical scales included attentional regulation (four items), effort regulation (five items), motivation (three items), time management (five items), and effort and time investment (two items). We were specifically interested to what extent students were able to manage their resources and adapt to emergency remote learning. Therefore, all items prompted students to think about the current situation and to retrospectively compare it to the situation before the change on a 5-point Likert scale from −2 (much less) to +2 (much more); i.e., the value of zero means no change. An example item is “In the current situation I get *much less/less/to the same extent/more/much more* distracted during self-study than before the crisis” (attentional regulation, reversed). See Supplementary Appendix A for all items.

#### Measures of Students’ Adaptation to Emergency Remote Education

To assess the extent to which the educational experience changed due to the shift to emergency remote education, we asked students to rate their overall experience with education before and during the pandemic on a scale from 1 to 10. Furthermore, we assessed how students’ engagement changed, as measured with four items on connectedness with peers, teaching staff, personal interest, and understanding on a 5-point Likert scale from −2 (decreased a lot) to +2 (grown a lot). An example item is “Since the beginning of the global health crisis, my sense of being connected with my fellow students has *decreased a lot/decreased/remained the same/grown/grown a lot*.” As indicator for the extent of adaptation to the situation, we further asked students to rate their mental well-being (“Compared to before the beginning of the global health crisis, how do you rate your mental well-being?”) on a scale from −2 (much worse) to +2 (much better).

#### Benefits and Difficulties

To assess potential benefits and difficulties of emergency remote learning, we asked two open-answer questions: “What did you like most during your online learning experience?” and “What did you dislike most during your online learning experience?”

### Procedure

Data collection took place in May 2020. The invitation to fill out the online questionnaire about their experiences after the shift to emergency remote education was sent in week 6 of the fifth course period to ensure that students had experienced the effects of the shift from face-to-face to emergency remote education for several weeks. At the start of the questionnaire, students provided their informed consent. Besides measures on students’ resource-management strategies, demographical variables, and indicators of adaptation, the questionnaire also asked more specifically about students’ experiences in tutorials, lectures, and online tool use. These data were only of interest for an internal report and not analyzed in this study. Students completed the questionnaire at home using their own digital devices. Completion of the questionnaire took approximately 15–20 min. This study was approved by the Ethical Review Board of the Faculty of Health, Medicine, and Life Science.

### Data Analysis

The quantitative data were analyzed using SPSS version 24 and SPSS AMOS. We examined the validity and reliability of the resource-management strategies questionnaire through confirmatory factor analysis using maximum likelihood estimation and calculation of Cronbach α value for each subscale used. We tested a five-factor correlated model. Because of the sensitivity of the χ^2^ statistic to sample size (Marsh et al., 1988), we used RMSEA (root mean square error of approximation) and SRMR (standardized root mean square residual) as overall model fit indicator, and the TLI (Tucker–Lewis Index) and CFI (comparative fit index) as comparative fit indices (Schermelleh-Engel et al., 2003). RMSEA analyzes the difference between the theoretical model and the population covariance matrix, with values between 0.05 and 0.08 indicating acceptable fit. The SRMR should be less than 0.05 to indicate good fit. The CFI compares the fit of the theoretical model to the fit of the independence model with all latent variables uncorrelated; values of >0.95 indicate acceptable fit. The TLI measures relative fit of the theoretical model compared to the independence model, with values between 0.95 and 0.97 indicating acceptable fit (Schermelleh-Engel et al., 2003).

To determine differences between students, we used an iterative partitioning method, the *k*-means cluster analysis, to classify students into groups based on their scores on attentional regulation, effort regulation, time management, motivation, and effort and time investment. Neither the group membership of the students nor the number of groups was defined beforehand. The aim of the *k*-means cluster analysis is to form homogeneous clusters by partitioning data in such a way that within-cluster variance is minimized and between-group variance is maximized. We followed the procedure outlined in Kusurkar et al. (2020). First, all clustering variables were standardized using *z*-scores. In the scale on attentional regulation, 17 cases were identified as outliers (*SD* >3) and excluded before further analyses as cluster analyses are highly sensitive to outliers. For the 1,800 participants included in the analyses, we tested 2-, 3-, 4-, 5-, and 6-cluster solutions. As an indication for model fit, we calculated the ratio between the between-clusters variance and the within-clusters variance for each solution using an analysis of variance (ANOVA) *F*-test. An acceptable cluster solution needed to explain at least 50% variance in the clustering variables scores. The optimal number of clusters was selected based on the explained variance, parsimony, and interpretability of the solution (Vansteenkiste et al., 2009; Kusurkar et al., 2020). As a validation procedure, we conducted a double-split cross-validation procedure to examine the stability of the chosen cluster solution (Vansteenkiste et al., 2009). We split the sample into two random subsamples and conducted the *k*-means cluster analysis again in these two subsamples. We computed Cohen κ with cluster membership of each subsample and the complete sample for checking the stability of the cluster solution. Subsequently, to explore the external validity of the cluster solution, we examined whether cluster profiles differed regarding their educational experience, engagement, and well-being using multivariate ANOVA (MANOVA) and *post hoc* comparisons using Bonferroni adjustments.

The qualitative data, i.e., the answers to the open questions, were thematically coded following a template approach (King, 2004) using several iterations. First, HH, SW, and WJ thematically coded the written answers to both questions, each of them being randomly assigned to a certain proportion of the data. Beginning with an open coding scheme, they continuously discussed, modified, and advanced the coding template until agreement was reached that the coding template covered all text sections. Second, we grouped the codes to the code groups of interest (i.e., the clustering variables and indicators of adaptation), see Supplementary Appendix B. Third, after finalizing the cluster analysis, we split the complete qualitative data set into subsets representing the identified clusters. SW and FB coded two subsets each, using the final coding template, while being blinded for the identity of the clusters. After a first round of coding, they discussed the codes and acted as second coder for each other’s subsets, respectively. See Supplementary Appendix C for an example. The data obtained from the thematic coding per cluster were summarized and compared to the data from the quantitative questionnaire for the same cluster. The entire research team was involved in this stage of triangulation of the cluster groups with the qualitative data. Relations and meaning of the themes were discussed in the research team, taking the analysis from the categorical to a conceptual level. ATLAS.ti qualitative software, version 8 (Scientific Software Development GmbH, Berlin, Germany), was used to analyze and manage the qualitative data.

### Reflexivity

The research team included an educational psychologist working as a Ph.D. candidate (FB); a cognitive psychologist working as professor in psychology (WW); an educational psychologist working as professor in education at FHML (AdB); and a physiologist and professor in education, working as scientific director of the FHML Educational Institute at Maastricht University (MoE). HH, SW and WJ have a background in social and educational sciences and were part of the project team for an internal report.

## Results

### Students’ Resource-Management Strategies During Emergency Remote Learning

To test the theoretical factor structure of the questionnaire on resource-management strategies, we conducted a confirmatory factor analysis using SPSS AMOS (Table 1). Confirmatory factor analyses showed an acceptable fit to the model according to the indices RMSEA, SRMR, and CFI; the TLI is just outside the acceptable ranges. χ^2^ was significant; for model acceptance, it should be non-significant. However, χ^2^ is highly dependent on sample size (Kline, 2005). Therefore, we chose to focus on RMSEA and the aforementioned model fit indices. The reliability of the scales provides further information regarding the model fit. All scales show acceptable to high internal consistency indicated by Cronbach α values ranging from 0.75 to 0.89. See the Supplementary Appendix for all items. Overall, these model fit indices indicate that the theoretical model of our adapted questionnaire has an acceptable fit. Internal consistency of the engagement scale was also acceptable with Cronbach α of 0.73.

**TABLE 1:** Model fit statistics for confirmatory factor analyses.

**Fit indices**	**Theoretical model**	**Threshold for acceptable fit**
χ^2^	806.21 (*p* = 0.000; *df* = 109)	
RMSEA (90% confidence interval)	0.060 (0.056–0.064)	0.05 < RMSEA < 0.08
TLI	0.948	>0.95
CFI	0.958	>0.95
SRMR	0.045	<0.05

Descriptive statistics and correlations between all variables measured are presented in Table 2. Students’ attentional regulation, referring to their ability to concentrate and deal with distractions, decreased the most in the current situation (mean = −0.87, *SD* = 0.86). Furthermore, students’ motivation (mean = −0.70, *SD* = 0.89), ability to manage their time (mean = −0.38, *SD* = 0.86), and ability to regulate their efforts (mean = −0.40, *SD* = 0.49) were perceived to decrease as well. The only positive value was related to effort and time investment (mean = 0.18, *SD* = 1.02), showing that students indicated that they put more time and effort in their self-study compared to the situation before the crisis.

**TABLE 2:** Means (and standard deviations) of and correlations between measured variables.

	**Mean (*SD*)**	**1**	**2**	**3**	**4**	**5**	**6**	**7**	**8**	**9**
(1) Attentional regulation	−0.87 (0.86)	–								
(2) Effort regulation	−0.40 (0.94)	0.567	–							
(3) Time management	−0.38 (0.86)	0.717	0.581	–						
(4) Motivation	−0.70 (0.89)	0.645	0.518	0.662	–					
(5) Effort/time investment	0.18 (1.02)	0.300	0.039^*ns*^	0.361	0.397	–				
(6) Well-being	−0.47 (0.93)	0.462	0.540	0.458	0.411	0.099	–			
(7) Engagement	−0.75 (0.65)	0.467	0.478	0.517	0.602	0.211	0.434	–		
(8) Educational experience before the crisis	8.02 (1.04)	−0.202	−0.134	−0.156	−0.160	−0.098	−0.117	−0.083	–	
(9) Educational experience during the crisis	5.72 (1.93)	0.423	0.424	0.498	0.535	0.202	0.365	0.573	0.121	–

*Scales for variables 1–7 range from −2 to 2; scales for variables 8 and 9 range from 1 to 10. All correlations are significant with p < 0.001, except when indicated with ns = non-significant*.

Correlations between all subscales of resource-management strategies were positive and statistically significant (*p* < 0.001), except for the correlation between effort regulation and effort and time investment (*r* = 0.039, *p* = 0.10). The highest correlations were found between attentional regulation and time management on the one hand and motivation on the other. A second correlational analysis was conducted between all subscales of resource-management strategies and the three indicators of adaptation: engagement, well-being, and overall educational experience during the crisis. All correlations were positive and significant (*p* < 0.001). The highest correlations were found between engagement and motivation, well-being and effort regulation, and educational experience during the crisis and engagement.

### Differences Between Students in Adapting to Emergency Remote Learning

**FIGURE 1:**
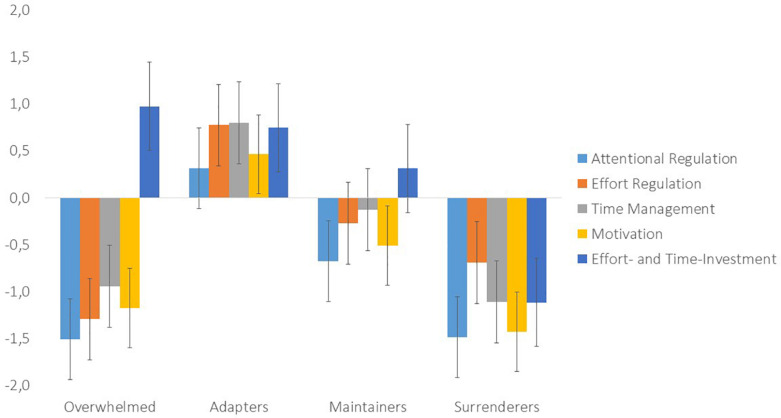
Four-cluster solution showing the adaptation in resource-management strategies per cluster. Data are presented as means with standard error, values of zero indicating no change.

In the cluster analysis, the four-cluster solution fitted the data best, based on the explained incremental variance, parsimony, and interpretability of the solution. The four-cluster solution explained 62.8% variance in the attentional regulation scores, 51.8% variance in the effort regulation scores, 65.7% in the time management scores, 56.4% in the motivation scores, and 60.1% in the effort and time investment scores. Figure 1 shows the four different groups identified based on the clustering variables, whereas Table 3 also presents their indicators of adaptation and demographic characteristics.

**TABLE 3:** Resource-management strategies, indicators of adaptation, and characteristics for each of the four identified clusters.

	**Overwhelmed (*n* = 393)**		**Adapters (*n* = 340)**		**Maintainers (*n* = 610)**		**Surrenderers (*n* = 457)**	
**Clustering variables**	**Mean**	**(*SD*)**	**Mean**	**(*SD*)**	**Mean**	**(*SD*)**	**Mean**	**(*SD*)**
(1) Attentional regulation	−1.51	(0.48)	0.31	(0.61)	−0.67	(0.53)	−1.48	(0.49)
(2) Effort regulation	−1.29	(0.56)	0.77	(0.68)	−0.27	(0.65)	−0.69	(0.72)
(3) Time management	−0.94	(0.50)	0.80	(0.48)	−0.12	(0.51)	−1.11	(0.52)
(4) Motivation	−1.17	(0.65)	0.46	(0.65)	−0.51	(0.56)	−1.43	(0.53)
(5) Effort/time investment	0.97	(0.65)	0.75	(0.66)	0.31	(0.71)	−1.11	(0.54)
**Indicators of adaptation**								
(6) Well-being	−1.01	(0.76)	0.37	(0.90)	−0.38	(0.77)	−0.77	(0.83)
(7) Engagement	−1.06	(0.54)	−0.12	(0.59)	−0.67	(0.53)	−1.06	(0.54)
(8) Educational experience score before the change	8.09	(0.92)	7.58	(1.28)	8.09	(0.97)	8.19	(0.92)
(9) Educational experience score after the change	4.82	(1.84)	7.36	(1.45)	6.07	(1.60)	4.79	(1.73)
**Characteristics**								
Females, *n* (%)	275	(70.0)	230	(67.6)	437	(71.6)	288	(63.0)
Males, *n* (%)	117	(29.8)	108	(31.8)	169	(27.7)	167	(36.5%
Bachelor, *n* (%)	336	(85.5)	287	(84.4)	501	(82.1)	408	(89.3)
Master, *n* (%)	57	(14.5)	53	(15.6)	109	(17.9)	49	(10.7)
Age in years, mean (*SD*)	21.3	(2.4)	21.7	(4.7)	21.3	(4.3)	20.8	(2.2)

*Scales for variables 1 to 7 range from −2 to 2; scales for variables 8 and 9 range from 1 to 10*.

The four clusters were labeled based on the reported resource-management strategies of the students in each cluster. The first cluster (*n* = 393) showed negative values in attentional regulation, effort regulation, time management, and motivation, showing that these students reported being less able to regulate their resources during emergency remote learning than in the situation before the crisis started. At the same time, this group reported investing more time and effort in their self-study. We therefore labeled the first cluster as the *overwhelmed*. The second cluster (*n* = 340) was characterized by positive values in all clustering variables, indicating that this group managed to regulate their attention, effort, and time better, and reported being more motivated than before the crisis. At the same time, they also mentioned investing more time and effort in their study. This group was classified as the *adapters*. The third group (*n* = 610) was characterized by negative values in attentional regulation and motivation and a relatively small increase in effort and time investment. This group changed their resource-management strategies the least compared to the other groups. We labeled this group as the *maintainers*. The fourth group (*n* = 457) was characterized by negative values on all scales, showing that they were heavily and negatively impacted by the situational change. On top of that and in contrast to the first group, they also reported investing less effort and time in their self-study. This group was therefore labeled as the *surrenderers*.

As a validation procedure, we conducted the double-split cross-validation procedure as outlined in Section “Materials and Methods (Data Analysis)”. This resulted in Cohen κ’s of 0.968 (*p* < 0.001) between the total sample and the first subsample and 0.954 (*p* < 0.001) between the total sample and the second subsample, indicating a stable cluster solution.

To evaluate the external validity of our resulting clusters, we conducted a MANOVA with cluster group as independent variable and well-being, engagement, and educational experience scores after the change as dependent variables. Results indicated a significant overall difference between the clusters, *F*(9,5385) = 99.07, *p* < 0.001, η^2^ = 0.14. Follow-up ANOVAs showed univariate effects for well-being, *F*(3,1795) = 202.7, *p* < 0.001, η^2^ = 0.25; engagement, *F*(3,1795) = 247.3, *p* < 0.001, η^2^ = 0.29; and educational experience scores during the global health crisis, *F*(3,1795) = 205.3, *p* < 0.001, η^2^ = 0.26. Bonferroni *post hoc* tests showed that the overwhelmed (cluster 1) and the surrenderers (cluster 4) did not differ significantly from each other regarding engagement, *p* = 1.00 [confidence interval (CI) = −0.09 to 0.10], and educational experience scores, *p* = 1.00 (CI = −0.27 to 0.33). All other cluster groups differed significantly in their mental well-being, engagement, and educational experience. The overwhelmed showed the highest decrease in well-being, and the overwhelmed and the surrenderers both showed the highest decrease in engagement and the lowest educational experience scores.

Additionally, we explored potential cluster differences regarding gender, bachelor’s or master’s degree level, and age. Gender distribution did not vary across the clusters, χ^2^(3) = 10.42, *p* = 0.108, but regarding bachelor’s or master’s degree level, χ^2^(3) = 10.7, *p* = 0.013, and age, *F*(3,1797) = 4.45, *p* = 0.004, η^2^ = 0.007, we did find differences. Inspecting the distribution across the clusters, proportionally more master students than bachelor’s degree students were in the maintainer profile. Bonferroni *post hoc* tests showed that students in the adapter cluster and the surrenderer cluster differed significantly in their age, with the adapters being older (mean_*age*_ = 21.7) than the surrenderers (mean_*age*_ = 20.8), *p* = 0.002 (CI = 0.24–1.61).

### Difficulties and Benefits Related to Emergency Remote Learning

We further investigated differences and commonalities between the cluster profiles in the qualitative analysis of reactions to the open-answer questions on benefits and difficulties related to emergency remote learning. In the following section, we will discuss the different profiles and their approach and adaptation to the situation, focusing on their attentional and effort regulation, motivation, time management, and effort and time investment. We illustrate key aspects with representative quotations from the written answers of participants. The profiles should not be interpreted as stable and fixed traits, but rather as reflecting the different adaptations and reactions of students to emergency remote learning.

#### The Overwhelmed

The overwhelmed were generally negative about emergency remote learning and the overall online learning experience. Concerning attentional regulation, students mentioned difficulties to concentrate and focus due to distractions at home, being online, and not having access to the library or other study facilities. Effort regulation was perceived as harder; spending long hours in front of a screen and internet connection problems were described as straining. Motivation was described to be negatively affected because of the lack of socialization and interaction with others; some students felt isolated and depressed. The lack of external structure and organization was also mentioned to be negatively influencing their motivation. Although the overwhelmed appreciated studying at home in a comfortable space and saving travel time, maintaining a daily routine became more difficult:

*“I dislike that we cannot do much meaningful discussions. I dislike that my internet connection is not helping. I dislike that my concentration from home is much worse than being at the [lecture] hall. I dislike that the libraries are closed and that I can’t find a place to study well.”*

The increased workload and stress were salient; students felt unsure about the online examinations and extra assignments and did not feel well-supported by the university:

*“All of this has to be understood in the context of not having proper schedules anymore. I guess some students have been able to adapt easily, but in my case, […] it’s as if there was no sense of being able to take breaks anymore. Lectures are becoming much longer than 2 h with the new materials given, and overall, I feel as if the study load has increased.”*

#### The Adapters

In general, the adapters appreciated the increased level of autonomy and self-directedness of the online setting. Students reported saving travel time, were better able to plan their days and make their own study schedule, and felt more in control of their day. Being able to watch the lectures at their own pace enabled students to check their understanding and study at times when they were more productive. This positively influenced their attentional and effort regulation, but also their time management:

*“I really loved the fact that all the lectures were recorded. I think it should be like that all the time. Because of this, I was able to skip a lecture and watch it later (at a later time when I was more productive). In this period, I have learned how to manage my time very well. I really like online education overall.”*

Nevertheless, the adapters missed the informal social contact with their tutors and peers and experienced collaboration with other students as more difficult online. These students also perceived that online examinations caused more stress and higher workload:

*“The only thing that I do not like about online education is the limitations that were imposed for online examinations, such as limited time, inability to change previous answers, higher intensiveness, more stress. […] There can be a lot of unexpected technical problems that we cannot be responsible for.”*

Many students in the adapter profile described themselves as either too shy to participate in normal, offline settings, or having long commuting times to and from university. The online setting enabled these students to save time and to study in a safe space at home, at their own pace:

*“It [online learning] took my anxiety away and made the uni experience much less stressful. It also lessened the pressure I was feeling, and I feel that my mental health has improved extraordinarily. Another great side effect was saving time that it took to go to and from university every day and has proven how much more efficient online communication is for me.”*

#### The Maintainers

In the maintainer profile, the experiences with online learning appeared to be more diverse. While appreciating the comforts of studying at home and saving time, the maintainers recognized the challenge of staying concentrated and motivated outside their regular study environment:

*“That you are no longer in this direct academic environment. Normally I would go to the library before or after, and I really need that because it has always been difficult to concentrate at home best. Of course you are online with everyone you would be in a tutorial with, and while that can also have benefits because you can do it comfortably from your home, it also took away some motivation from me for sure.”*

Students further missed the direct contact with their tutors and peers and criticized education to be less interactive and effective than usual. Nevertheless, many maintainers showed understanding for the uniqueness of the situation and appreciated the communication of the university and guidance by tutors and course coordinators:

*“In general, I am not too excited about online education, but the flexibility it brings to follow education from wherever is sometimes nice. I appreciate how hard the university is trying to communicate and develop.”*

#### The Surrenderers

Comparable to the overwhelmed, students in the surrenderer profile described their general educational experience as negative; they experienced great difficulties with attentional regulation, motivation, and time management. Some students in this profile also mentioned an increase in stress and workload, similarly to the overwhelmed. Most students mainly experienced a decrease in their motivation due to the lack of interaction with others and their general educational experience, which might explain the drop in their effort and time investment:

*“The absolutely most essential thing about university is getting excited about what you learn. I easily get excited for what I learn. This period is different. Lacking friends and staff members all around me to bump into and exchange ideas with was the stimulating thing at university. Now that is completely missing, and I wake up wondering why I am studying at all. This lack of a common area and extrinsic motivation brings down the quality of what everyone contributes to PBL significantly.”*

At the same time, they did not invest as much time and effort in their study as the overwhelmed. Students in this profile perceived the increase in self-direction and autonomy as a burden. While they appreciated saving time and studying at home in a comfortable environment, the surrenderers had difficulties to regulate their resources during self-study:

*“My motivation significantly decreased. I am also studying way less than I would usually do. Though I never missed any activities before the COVID-situation, now I no longer follow my timetable and leave the lectures for later.”*

Furthermore, many students in the surrenderer profile felt a mismatch between their PBL learning experience in an on-site setting as compared to the online setting. They were, moreover, critical about online learning in general:

*“Online learning is not working. Quality of education provided by the university through online learning was significantly less. This was not because tutors were not prepared, but because online learning does not fit PBL and most courses.”*

In summary, during emergency remote learning, all students faced similar challenges, but students of the different cluster profiles coped with these challenges differently. Students of all profiles missed the personal contact with teachers and peers. The reduced collaboration and interaction negatively influenced their motivation. All students saved travel time, but the adapters appreciated the increase in autonomy and self-directedness, being able to study at their own pace. The overwhelmed and surrenderers struggled most to manage their time, attention, and efforts effectively.

## Discussion

The aim of this study was to examine how and to what extent university students adapted to emergency remote learning in the context of the COVID-19 pandemic. Using a mixed-methods approach, we first investigated how the sudden shift from face-to-face to emergency remote education influenced students’ self-regulated learning, specifically focusing on their resource-management strategies. We administered a questionnaire on students’ resource-management strategies during emergency remote learning and on indicators of (un)successful adaptation: general educational experience, engagement, and mental well-being. Our findings indicate that, in general, students experienced more difficulties in managing their time and regulating their attention and efforts and reported being less motivated than before the shift to online education. Furthermore, on average, students mentioned investing more time and effort in their self-study. In line with the difficulties in managing their resources, students experienced a decrease in their mental well-being and engagement with their studies, and their general educational experience dropped significantly.

Given the uniqueness of the situation and individual differences in self-regulated learning (Dörrenbächer and Perels, 2016), we assumed that students would differ in their abilities and approach to adapt to emergency remote learning. With the use of a person-centered approach (Kusurkar et al., 2020), we identified four adaptation profiles and labeled them according to the reported changes in their resource-management strategies: the overwhelmed, the adapters, the maintainers, and the surrenderers. These profiles allowed for a differentiated perspective on the ways students adapted to emergency remote learning. Most students were classified as maintainers (*n* = 610, 34%). Although their attentional regulation and motivation decreased compared to before the crisis, students’ ability to regulate their efforts and to manage their time, as well as their time and effort investment, did not change significantly. Both the overwhelmed (393 students, 22%) and the surrenderers (457 students, 25%) experienced difficulties to adapt to emergency remote learning. These students reported being less motivated and less able to concentrate, manage their time, and regulate their efforts. At the same time, the overwhelmed reported investing more time and effort in their self-study, whereas the surrenderers showed a decreased investment of time and effort in self-study activities. Both groups rated the educational experience as worse than before the crisis, while their engagement and well-being dropped, indicating that students in these profiles were unsuccessful in adapting to emergency remote learning. The fourth subgroup of students, classified as the adapters (340 students, 19%), can be considered as the group that was most adaptive to the new situation: these students reported to be more motivated and better able to regulate their attention, effort, and time than before. At the same time, this group also invested more time and effort in their self-study. Students in the adapter profile reported even a slight increase in their well-being, whereas their educational experience stayed relatively stable compared to before the crisis.

Some students struggled more on time and effort investment, whereas others struggled more regarding attention and motivation. This multidimensionality of resource-management strategies suggests a tailored support approach for students. While the surrenderers might benefit from more structure and social interaction, the overwhelmed might need more support on stress management. These results further support prior person-centered research on self-regulated learning and motivational profiles by identifying different subgroups ranging from high to low adaptability regarding resource-management strategies (e.g., Vansteenkiste et al., 2009; Broadbent and Fuller-Tyszkiewicz, 2018). However, in contrast to the online students in the study by Broadbent and Fuller-Tyszkiewicz (2018), most students in our sample related to non-adaptive profiles. This stresses the difference between students who actively chose for online learning and students who were forced to study in an emergency remote learning setting. The latter group might need more guidance and support in an online learning environment.

To gain a deeper understanding of the differences between the profiles, we analyzed the answers regarding experienced difficulties and benefits of emergency remote learning for each profile. While the aforementioned differences between the profiles were clearly represented in the open answers, similarities were noted as well. Students of all profiles appreciated the recorded online lectures and being able to study at home. However, the quality of interaction and level of active learning while studying at home differed. While the adapters mentioned being able to study in their own pace and play and pause the online lectures to monitor and control their understanding, the surrenderers rather appreciated the comfort of staying at home and not having to travel to university. This finding illustrates the difference between students in their ability to effectively apply self-regulated learning strategies, and resource-management strategies in particular. Not having access to learning facilities, such as the library, was mentioned to be a clear disadvantage and hampering students’ attention and effort regulation. Students in the surrenderer profile, for example, mentioned to be highly reliant on the library to study and distractions at home hindered their resource management.

With the majority of students not being able to take advantage of the higher levels of autonomy associated with emergency remote learning and given the importance of these skills for academic achievement in online learning (Puzziferro, 2008), it is necessary to support such students in their self-regulated learning. Future research could investigate, for example, whether prompts included in online lectures can support students in the low-adapting profiles to monitor and control their understanding and enhance their attentional regulation and motivation (Lehmann et al., 2014). The difference between students in their ability to adapt to emergency remote learning might be further explained by personality factors. Some students in the adapter profile mentioned to be shy; they felt safer participating in online education. More extraverted students, on the other hand, might suffer more from isolation and reduced collaboration in education. Furthermore, consistent with previous research, older age appeared to be related to a higher adaptive profile (Johnson, 2015). Age might be a proxy for more experience in higher education and therefore for a better ability to self-regulate. Tailored support, depending on the ability to adapt to emergency remote learning, could be beneficial (Dörrenbächer and Perels, 2016). While this study helped identifying the different groups of learners, further research into the specifically applicable interventions is needed. It would be worthwhile to examine in a longitudinal study whether these different adaptation profiles are stable. In that case, it would be of interest to measure students’ resource-management strategies at the beginning of an online course to provide tailored support and mentoring during the online learning experience.

Students of all four profiles reported having missed the social contact and interaction with their teachers and peers. The reduced collaboration was described as less motivating compared to face-to-face education. Online communication and collaboration, especially in tutorials, were experienced as more straining due to long screen times and the lack of non-verbal communication. These findings resonate with the challenges of blended online learning environments, such as increased feelings of isolation and disinterest, and students feeling alienation and isolation in online learning (McInnerney and Roberts, 2004; Rasheed et al., 2020). How to facilitate collaboration in online education and address students’ isolation are important questions for future research. For example, as suggested by McInnerney and Roberts (2004), social interaction in the online environment could be enhanced through increasing the use of synchronous communication and dedicating time to form a sense of community, for instance, by starting synchronous contact with low-stakes learning tasks, using visual cues to guide learners’ attention and prioritize tasks and resources that require low bandwidth to reduce internet connection problems (Green et al., 2020). In order to create and maintain academic communities and relationships, it is necessary to scaffold communication and collaboration carefully and to combine both synchronous and asynchronous contact with teachers and peers (Nordmann et al., 2020).

The fact that students in the current study were used to a highly interactive and collaborative educational format (PBL) may have contributed to the aforementioned difficulties to adapt to a less collaborative format. Students that had initially chosen to study in a highly collaborative setting were now forced to study in a highly autonomous learning environment with online contact only. Students in the low-adapting profiles (surrenderers and overwhelmed) often mentioned a general mismatch between their online and on-site experiences with PBL. Their negative attitude was also reflected in their general educational experience scores. In a transition from face-to-face to online education, it seems therefore important to guide the transition and align the expectations of students and teachers toward the online format (Kebritchi et al., 2017; Nordmann et al., 2020). Most students also experienced increased workload and invested more time in their self-study. They often mentioned examination-related stress and uncertainty about assignments and online proctoring as a reason. Managing the expectations of students regarding the online format and the way examinations are structured through more guidance and communication could alleviate stress and experienced workload.

This study has several limitations. First, the generalizability of our results might be limited given the PBL context in which the participants of this study were studying. Students were used to participate in small, discussion-based tutorial sessions. In the crisis situation, tutorial sessions continued online, with less active discussions and a lack of non-verbal communication, which may have had a larger effect on students’ self-regulated learning strategies than in a more traditional curriculum. Furthermore, the response rate of 10.5% was rather low. Given that non-respondents may be students who have experienced significantly more or less difficulties due to the pandemic, this might have biased the results. However, the composition of the sample was highly diverse, with students of all faculties and including bachelor’s and master’s degree students of different years and may therefore be considered as relatively representative for this university.

Second, the measurement of students’ adaptation to the situation was based on self-report and may have been altered by retrospective bias (Stone and Shiffman, 2002). As respondents were asked to compare the current situation to the situation before the shift to emergency remote education, no conclusions can be drawn regarding the general level of self-regulation; the findings only provide information about the level of adaptations to the change. Moreover, we adapted existing questionnaires on online self-regulated learning to capture the level of self-regulation during a change to emergency remote learning. We specifically focused on students’ resource-management strategies given the increased relevance of these strategies during the crisis and did not assess students’ cognitive and metacognitive strategies. Future research on how students adapt to online learning could include all aspects of self-regulated learning to generate a complete picture.

## Conclusion

While the emergency part of emergency remote learning may not be as emergent anymore and universities might go back to full face-to-face education as soon as possible, online education and remote education are likely to remain part of future educational formats. The current study sheds light on how students adapted to online education in the context of a crisis. While many students experienced difficulties to manage their resources and engage in self-regulated learning, different profiles of adaptation emerged: the overwhelmed, the surrenderers, the maintainers, and the adapters. These profiles may serve as framework for future research on tailored interventions to support students adapting to online and remote education. Important aspects entail the focus on facilitating online collaboration and socialization to conquer feelings of isolation, guiding attentional and effort regulation during self-study, and managing students’ expectations about online learning.

## Data Availability Statement

The datasets generated for this study are not readily available because informed consent signed by participants stated that data were only accessible to the authors of this study. Requests to access the datasets should be directed to FB, f.biwer@maastrichtuniversity.

## Ethics Statement

The studies involving human participants were reviewed and approved by the Ethical Review Board of the Faculty of Health, Medicine, and Life Sciences, Maastricht University, the Netherlands (approval number: FHML-REC/2020/065). The patients/participants provided their written informed consent to participate in this study.

## Author Contributions

All authors were responsible for the design of the study. FB performed analysis of the quantitative data, in close collaboration with WW. HH, WJ, SW, and FB performed analysis of the qualitative data, in close collaboration with the other authors. FB drafted the article, incorporating edits, and feedback from all other authors (WW, AB, ME, HH, WJ, and SW). All authors made a substantial contribution to the interpretation of the data for this work.

## Conflict of Interest

The authors declare that the research was conducted in the absence of any commercial or financial relationships that could be construed as a potential conflict of interest.
