# Supplementary material for: Changes and Adaptations: How University Students Self-Regulate Their Online Learning During the COVID-19 Pandemic
Source: Front Psychol. 2021 Apr 23;12:642593. doi: 10.3389/fpsyg.2021.642593 (PMC8103204; doi:10.3389/fpsyg.2021.642593)
Supplement: Supplementary file 1 [file Table_1.docx]

**Appendix A**

*Questionnaire on Resource Management Strategies, with Means (M) and Standard Deviations (SD) per item and Internal Reliability of all Scales (Cronbach’s Alpha)*

|  |  | M | SD | α |
| --- | --- | --- | --- | --- |
| AR1 | In the current situation, I encounter __ distractions in my study environment than before the crisis (reversed) | -0.90 | 0.99 |  |
| AR2 | In the current situation, my mind wanders __ during my self-study than before the crisis. (reversed) | -0.89 | 0.96 |  |
| AR3 | In the current situation, I get __ distracted during self-study than before the crisis. (reversed) | -0.91 | 0.97 |  |
| AR4 | In the current situation, I can concentrate __ during self-study than before the crisis. | -0.80 | 1.04 |  |
| **AR** | **Attentional regulation (scale)** | **-0.87** | **0.86** | **.89** |
| ER1 | In the current situation, I feel __ exhausted after a self-study session than before the crisis. (reversed) | -0.54 | 1.05 |  |
| ER2 | In the current situation, I am __ able to manage my energy during a study day than before the crisis. | -0.43 | 1.13 |  |
| ER3 | In the current situation, I am __ able to relax in my free time than before the crisis. | -0.24 | 1.27 |  |
| **ER** | **Effort regulation (scale)** | **-0.40** | **0.94** | **.75** |
| ET1 | In the current situation, I put __ effort in my self-study sessions than before the crisis. | 0.12 | 1.07 |  |
| ET2 | In the current situation, I put __ time in my self-study sessions than before the crisis. | 0.23 | 1.15 |  |
| **ET** | **Effort and time-investment (scale)** | **0.18** | **1.02** | **.82** |
| M1 | In the current situation, I experience __ motivation to prepare for tutorial meetings than before the crisis. | -0.71 | 1.05 |  |
| M2 | In the current situation, I experience __ motivation to keep up with the course program than before the crisis. | -0.65 | 1.04 |  |
| M3 | In the current situation, I experience __ motivation to prepare for exams than before the crisis. | -0.75 | 1.02 |  |
| **M** | **Motivation (scale)** | **-0.70** | **0.89** | **.83** |
| TM1 | In the current situation, I choose specific times when I study effectively __ than before the crisis. | -0.09 | 1.05 |  |
| TM2 | In the current situation, I find it __ hard to stick to a study schedule than before the crisis. (reversed) | -0.65 | 1.06 |  |
| TM3 | I currently have an effective study routine that has helped me in the past. | -0.20 | 1.07 |  |
| TM4 | I find it difficult to adapt my study routine to the current situation. (reversed) | -0.44 | 1.22 |  |
| TM5 | In the current situation, I am able to continue my study routine as before. | -0.52 | 1.16 |  |
| **TM** | **Time-management (scale)** | **-0.38** | **0.86** | **.83** |

*Note*. AR = Attentional regulation, ER = Effort regulation, ET = Effort- and time-investment, M = Motivation, TM = Time management; N = 1800.

| **Theme group** | **Themes per question** | |
| --- | --- | --- |
|  | What did you like about online education? | What did you dislike about online education? |
| Motivation | - Increased autonomy and self-directedness | - Lack of personal contact |
|  | - Assessment structure (formative, asynchronous) | - Less motivated / engaged/ connected |
|  |  | - Less collaboration |
| Time management | - More flexibility: Studying at own pace | - Lack of structure - Self-directedness |
|  | - More flexibility: study at home - More flexibility: save time (less travel time) | - Workload and stress increased |
| Effort regulation | - Comfort of study at home, getting more rest | - Too much screen time |
| Attentional regulation | - Online lectures: easier to follow/ able to re-watch/ check understanding - Concentration: easier to follow shorter tutorials / lectures | - Connection issues - Concentration: cannot focus - Concentration: no access to study facilities |
|  |  |  |
|  |  |  |
| Time and Effort investment |  | - increased workload |
| Educational Experience | - positive attitude towards online learning | - negative attitude towards online learning |
| Wellbeing | - feeling at ease / less stressed/ more rest | - feeling stressed, negative mood |

**Appendix B**

*Coding scheme per theme group*

**Appendix C**

*Coding scheme example for Cluster 4 ‘The surrenderers’*

| **1. What did you like about online education?** | **2. What did you dislike about online education?** | **Themes** |
| --- | --- | --- |
| “I was able to be at home and I didn't need to travel back and forth. I could also adjust the times when I wanted to watch a lecture or practical. It enabled me to have more time to eat and to take care of myself, I was less in a rush.” | “My motivation significantly decreased. I am also studying way less than I would usually do. Though I never missed any activities before the Covid situation, now I no longer follow my timetable and leave the lectures for later.” | 1.More flexibility: Save time (less travel time)  1. More flexibility: Study at home  1. Comfort of study hat home  2. Less motivated/engaged/ connected  2. Lack of structure  2. Time-investment |
| “Nothing. Literally nothing. I hated the whole experience. I hate having to interact with people through screens for extended periods of time, and especially on academic related subjects. I cannot stress how much I liked no aspect whatsoever of this whole thing.” | “The fact I cannot interact with other people naturally. The fact I have to go through a screen to interact with anyone. if I wanted an online course on anything, I would have gotten an online degree. I hated this learning experience. Whilst I do understand the necessity of online learning to an extent, I don't believe this is the crux of your question. This was a miserable experience.” | 2.too much screen time  2. less motivated/engaged/ connected  2. negative attitude towards online learning |
| “All the lectures and material were online so people could go at their own pace. Recording lectures should be a normal practice at UM. Attendance was not mandatory (this is very important)” | “Feeling disconnected from the class, not getting to know the teacher nor the fellow students.” | 1.More flexibility: study at own pace / study at home  2. lack of personal contact  2. less engaged/connected |
| “More peaceful and less stress” | “No motivation, feeling alone, hard to get into studying even if you make a planning or want to work you just can't, missing friends” | 1.More flexibility: save time / study at home  2. Less motivated / engaged/ connected  2. Lack of personal contact |
